# Square Pegs in Round Holes: How Strict Policies and Standardization of Mental Health Treatment Practices Complicate Youth Care Seeking

**DOI:** 10.1007/s10826-026-03273-6

**Published:** 2026-02-12

**Authors:** David A. A. Miller, Scott T. Ronis

**Affiliations:** 1 Department of Psychology 38 Dineen Dr, Keirstead Hall, University of New Brunswick, Fredericton, Canada; 2 School of Social Sciences, Education and Social Work, Queen’s University Belfast, Belfast, Northern Ireland, UK

**Keywords:** Youth, Mental health, Treatment, Standardization, Policy

## Abstract

Public mental health practices related to treatment assignment and delivery are increasingly becoming standardized, yet little is known about which standardized practices affect youth treatment seeking. Although standardization introduces consistency in procedures, diagnoses, and treatment, and is considered beneficial to institutions and individuals for a variety of reasons (e.g., reliability of administration, predictability of outcomes), it often reduces individuals to diagnostic or symptom-specific classifiers and disproportionately affects care seeking among youth due to their marginalized position in care delivery. We conducted semi-structured interviews with 34 youth, ages 10 to 21 years, as part of the Atlantic Canada Children’s Effective Service Strategies Mental Health (ACCESS-MH) project to assess the impact of standardized care practices on youth mental health care seeking. Based on analyses using Psycho-Social Ethnography of the Commonplace (P-SEC) methodology, findings highlight the struggle of marginalised individuals (i.e., youth in need of mental health services) when trying to navigate mental health systems. Findings indicate that standardization of care can act as a barrier for youth due to shortcomings in the appropriateness and timeliness of interventions, and that treatment monitoring and the use of modalities less reliant on categorical diagnosis could help to address the negative impact. Recommendations related to policy change [e.g., treatment monitoring, the use of modalities less reliant on categorical diagnosis] are discussed.

## Highlights

Youth hold a marginalized position in mental health care delivery due to an overreliance on caregivers and providers.

Youth perspectives provide valuable insight into the ways in which standardized practices complicate their care seeking.

Youth identify overreliance on diagnosis, iatrogenesis, and inadequate follow up/monitoring stemming from standardization.

Increased monitoring and youth collaboration, along with transdiagnostic approaches, would address identified complications.

The standardization of mental health treatment practices and policies is a by-product of the field’s deliberate shift toward aversion from legal risk and prioritizing evidence-based and evidence-informed care (Drake et al., 2001; Gregory et al., 2023). Although standardizing policies related to treatment access offers clear benefits for both providers and service users (Cook et al., 2017; Drake et al., 2001), it also carries the risk of becoming a barrier to access due to an increased reliance on rigid, categorical criteria (e.g., demographics, diagnostic classification) to inform treatment planning (Patalay & Fried, 2021). Indeed, even proponents acknowledge that these practices may result in less-than-ideal support (Patalay & Fried, 2021). This is especially salient for youth, who often face systemic barriers due to their marginalized position in the mental health system (Birleson & Vance, 2008; de Haan et al., 2013). Research shows that young people experience unique mental health treatment journeys compared to their adult counterparts, with increased dependence on others to navigate critical aspects of care (e.g., symptom recognition, treatment adherence, financial support; Birleson & Vance, 2008; de Haan et al., 2013; Tilleczek et al., 2017). Because approximately 20% of youth experience clinically impairing mental health issues with only one in five receiving care (Gandhi et al., 2016), it is crucial to examine how restrictive policies and standardized practices may play a complicating role in treatment access.

Despite growing concerns about access (see Kourgiantakis et al., 2023), existing research often focuses on the impact of standardized protocols on institutional outcomes (e.g., length of stay, attrition, readmission) or clinical effectiveness (de Haan et al., 2013; Fontanella, 2008; Mohamed et al., 2018; Mulder et al., 2022), with limited attention to potential iatrogenic effects. This gap may be due, in part, to the literature generally framing standardized practices as beneficial (Cook et al., 2017; Drake et al., 2001). It also reflects a predominant reliance on administrative data, which obscures individual experiences. The current study addresses gaps by capturing youth perspectives on strict policies and standardized practices in relation to access and treatment experiences through a qualitative approach. By doing so, the data are positioned to inform practical changes to mental health care systems that would address barriers for youth seeking treatment. Understanding how standardized practices and policies are implemented and experienced is essential to advancing more equitable and responsive mental health systems.

## Standardized Practices and Policies

Standardized practices in the context of public inpatient and outpatient care encompasses a range of policies and protocols that serve to organize and routinize the assessment and treatment of patients (Martin et al., 2011). For the purposes of this study, standardized practices are operationally defined as the use of predetermined criteria (e.g., demographics, diagnoses, symptom acuity) to dictate an individual’s appropriateness for treatment or the treatment options available to them. Research suggests there are several benefits to care providers and institutions stemming from standardized and often strict policies, the majority of which pertain to management of individual treatment (e.g., effectively determining treatment plans in a timely manner), maintaining a competent workforce (e.g., targeted training for staff in commonly used core assessment and treatment skills), and continuity of care (e.g., regimented care plans with planned pathways based on symptom progression; Drake et al., 2001; Gregory et al., 2023).

Although treatment path assignment practices can vary among institutions, most typically rely on standardized policies emphasizing diagnostic criteria (e.g., the *International Classification of Diseases [ICD]*, the *Diagnostic and Statistical Manual of Mental Disorders [DSM]*; American Psychiatric Association, 2013; World Health Organization, 2003). Based on these and other relevant factors (e.g., age, gender, risk level, degree of impairment), individuals are triaged into specific treatment plans or paths in accordance with their difficulties. Treatments are often evidence based or evidence informed (e.g., practices established as efficacious and effective for specific presentations through rigorous evaluation), dictated by diagnosis, and delivered in manualized or structured form to increase reliability among providers and treatment settings (Cook et al., 2017; Drake et al., 2001). Despite operational advantages, many of these interventions are structured to meet needs in the general population and address core symptoms and related deficits of target disorders with minimal consideration of extraneous factors (e.g., environmental contributors, comorbid symptoms; Martin et al., 2011). As such, they often lack transferability and tend to be overly reductive (Cook et al., 2017; Patalay & Fried, 2021), which may result in individuals not receiving treatment that best aligns with their broader needs. This is particularly problematic among youth, who exhibit developmentally distinct mental health challenges and may have difficulty navigating the available services if those services that are offered are not appropriate (Martin et al., 2011). Further, evidence suggests that standardized practices are typically based in a Western worldview and, thus, may not meet the needs and values of the approximately 20% of Canadian youth who identify as visible minorities (Erbach, 2022).

## Youth Considerations

Youth hold a unique position in mental health treatment seeking that may not be well served by restrictive approaches. First, youth, compared to adults, are disproportionately disconnected from the decision-making process inherent in mental health care (de Haan et al., 2013). Although youth and adults may experience similar policies and protocols that indicate recommended treatments, youth often lack the legal autonomy and capacity to consent to, or make informed decisions about, their care (Birleson & Vance, 2008). This is concerning as requirements that youth rely on parents or guardians for consent may lead them to avoid seeking support. Further, they hold little sway in influencing higher-order policy and institutional factors (de Haan et al., 2013). Second, youth are objectively poor advocates of their own mental wellness and need for intervention, instead relying on parents, guardians, or service providers for symptom identification, treatment adherence, and follow-up (Birleson & Vance, 2008; de Haan et al., 2013). Third, prominent lifespan psychopathology models (see Mash & Wolfe, 2018) indicate that mental illness predominantly presents in adolescence, with multiple trajectories and high comorbidity among diagnoses. This diverse developmental course and symptom presentation make it challenging to conceptualize problems among youth based solely on a standardized, categorical diagnosis (Astle et al., 2022; Kessler et al., 2005). Taken together, these limitations suggest that youth experience strict policies and standardized practices as ill-fitting and poorly aligned with their lived realities.

### Mentally Ill Youth as Marginalized Individuals

The unique power imbalance that youth experience in relationships with their guardians, service providers, and care organizations (see Birleson & Vance, 2008; de Haan et al., 2013) limits the scope of influence youth have in dictating their mental health journeys. It also places them on the margins of care delivery. Marginalization has been identified by the American Psychological Association (2020) as individuals or groups possessing distinctive qualities that result in barriers to gaining full acceptance into the larger group. This framing of youth as a marginalized group offers a critical lens through which their treatment experiences can be better understood. Thus, examination of youth perspectives on mental health treatment requires acknowledgement of their unique marginalized position within the overarching care system.

## Theoretical Framework—Psycho-Social Ethnography of the Commonplace (P-SEC)

Conceptualization of youth difficulties followed the Psycho-Social Ethnography of the Commonplace (P-SEC; Gouliquer & Poulin, 2005) framework. The P-SEC framework has theoretical foundations in feminist standpoint epistemology (i.e., wherein individuals possess knowledge unique to their objective account of the world; Harding, 2004). It also borrows from a number of psychological and sociological theories, including institutional ethnography (Smith, 2005) and schema theory (Beals, 1998; Bem, 1993; Rumelhart, 1980). Extrapolating from the underpinning theories, the P-SEC framework proposes that individuals in powerless or marginalised positions are uniquely situated to describe an accurate account of the real world, due to their understanding of their own worldview and the worldview of those who oppress them. Further, P-SEC posits that marginalized individuals are frequently subjected to institutional “relations of ruling” (i.e., unique social orders and conventions), despite the discordance with their marginalized status. As a result, marginalized individuals are often forced to facilitate their own oppression by participating in these orders and conventions (Smith, 2005). This process of self-facilitated oppression often creates complications for individuals, which require them to adapt and cope with situations outside their control as they attempt to resolve the disparity between their own needs and what is required of them by the institution. Doing so alters their understanding of the world, which results in modifications to existing schemata to make sense of their unique reality (Beals, 1998; Bem, 1993; Rumelhart, 1980). The complications individuals experience due to these orders and conventions are conceptualized within P-SEC as Organizational Moments. An analysis of Organizational Moments allows for the voices of marginalized individuals to be brought to the forefront in a critical examination of the Organization and its practices (Gouliquer & Poulin, 2005). Informed by this framework, the present study was designed to center youth voices and document how they interpret and respond to institutional barriers.

### Study Purpose

The goal of the current study was to explore whether standardizing policies related to access and treatment complicated youth mental health journeys. A qualitative approach was used to offset the existing preponderance of administrative data in current literature to provide detailed and contextual understanding of youth experiences in care seeking. Analysis and interpretation of youth interviews were used to answer four research questions (RQs):

1. How do organizational practices, policies, and protocols complicate youth mental health care seeking?
2. How do youth cope with barriers if and when encountered?
3. In what way do youths’ conceptualization of their difficulties change in response to barriers?
4. How are youth understandings of mental health treatment shaped by their experiences?

## Method

### Participants

Thirty-four interviews were conducted from 2014 to 2016 with youth 10 to 21 years old (*M* = 16.44 years, *SD* = 2.46) across Atlantic Canada (i.e., Prince Edward Island, New Brunswick, Nova Scotia, and Newfoundland and Labrador; see Table 1 for demographic information). All youth had a current diagnosis of, or identified as, experiencing at least one common child or youth mental health condition (i.e., autism spectrum disorder, conduct disorder, eating disorders, anxiety, depression). Interviews were conducted as part of a larger study with youth, caregivers, and service providers examining barriers and facilitators of access to mental health services (i.e., the Atlantic Canada Children’s Effective Service Strategies in Mental Health project [ACCESS-MH]; ACCESS-MH, 2014). Qualitative maximum variation sampling rules dictated selection of participants to reach saturation (Morse, 2000). Pseudonyms were assigned to all participants, with unique pseudonyms (i.e., discontinuous pseudonyms) for each quote to further ensure anonymity. In addition, minor inconsequential details were altered as needed (e.g., substituting one extra-curricular activity for another) across interviewees’ accounts to ensure confidentiality.

**Table 1:** Participant Characteristics

Characteristic	Total *N* = 34 (%)
Gender *n* (%)	
Male	12 (35.3)
Female	22 (64.7)
Province of origin	
Prince Edward Island	14 (41.2)
New Brunswick	7 (20.6)
Nova Scotia	4 (11.8)
Newfoundland and Labrador	9 (26.5)
Reported diagnosis made by a mental health professional	26 (76.5)
Anxiety	19 (55.9)
Depression	16 (47.1)
Autism spectrum disorder	3 (8.8)
Eating disorder	7 (20.6)
Perceived familial financial standing (*My family…*)	
* Does not have enough money*	7 (20.6)
* Has enough money*	24 (70.6)
* Has lots of money*	3 (8.8)
Currently in school	
Yes	30 (88.2)
No	4 (11.8)
Currently employed	
Yes	15 (44.1)
No	17 (50.0)

### Materials and Procedure

Recruitment strategies consisted of poster advertisements in public spaces related to youth mental health.

(e.g., service providers’ offices, Canadian Mental Health Association offices, school guidance counselors’ offices) and communication with key mental health service representatives (i.e., psychiatrists, physicians, hospital administrators) to post advertisements in their spaces. An effort to reduce potential selection bias was made by allowing youth to self-refer to the study via the posters. Snowball sampling was also used. All participants provided written informed consent or assent (for those youth under the age of 18) prior to beginning their interview session, with legal guardians providing consent when required for youth below the age of majority. The consent process consisted of a detailed explanation of the study’s purpose, the data collection process, and the participant’s right to withdraw participation at any time. Interviews were conducted in person with participants by student researchers enrolled in a graduate-level applied health research program. Youth were prompted to provide demographic information (e.g., age, gender, ethnicity, first language, country of origin, employment status, diagnosis), and their perceptions of self, life, and experience as they related to their mental health journeys (Minichiello et al., 2008). Journey mapping was used to address three consistent challenges inherent in collecting youth perspectives of their mental health experiences: (1) journeys are often fractured and non-linear, (2) various individual personal and systemic influences exist, often paradoxically, and (3) journeys often begin long before initial interventions, with schools playing a crucial role at various points (Tilleczek et al., 2017). This practice allowed youth to create a visual representation of their narrative to aid in storytelling only, and, as such, were not directly included in the analysis.

Rigor for the present study was achieved through practices related to credibility (i.e., using participants’ own words in describing their experience), transferability (i.e., collecting data through standardized methods across a broad geographic region), dependability (i.e., identifying youth as a marginalized group within public mental health based on available empirical data), and confirmability (i.e., allowing youth to lead the interview process). Youth and guardians were also provided with a list of community mental health resources following completion of their interviews. Ethical approval for data collection was obtained from the five principal Atlantic Canadian institutions involved in the ACCESS-MH project as stipulated according to Helsinki Declaration guidelines.

### Data Analysis

Following the PSEC framework, we conducted a three-step analytic process: (1) interview transcription and broad thematic coding to identify complications (i.e., Organizational Moments) related to typical practice; (2) identification of associated themes indicating the ways in which the Organization benefits, the ways in which individuals cope with the experience, and any associated changes to individuals’ cognitive schemata; and (3) solicitation of the organization’s reactions and comments regarding the marginalised individuals’ concerns. The administrators’ comments are considered an official response and included in the summary of findings if provided.

### Organizational Moments

Organizational Moments are formal or informal practices, policies, rules, or events that occur regularly to meet the needs of an organization. They must also shape the activities and thoughts of a marginalised group (e.g., youth with mental illness) and complicate their lives (Gouliquer & Poulin, 2005). For the purposes of data analysis, Prince Edward Island (PEI) public mental health treatment services were identified as the organization of focus (referred to hereafter as the Organization). The selection of PEI public mental health services as the organization of focus was done for efficiency and because the majority of interviews were conducted with youth living in PEI (i.e., 41.2%), with findings considered to be largely transferrable to other places across Atlantic Canada. Indeed, Atlantic provinces are similar in their representative populations, geography, and health care systems (Hayward & Colman, 2003; Latham, 2012), and youth commonly receive services in other Atlantic provinces due to limitations in the availability and accessibility of services in their home provinces (Latham, 2012).

## Results

**Table 2:** Organizational Moment and Supporting Themes

Organizational Moment	Complications	Schemata	Coping
*Standardization of Practices*	1. Meeting criteria^1^ 2. Partial help^1,3^ 3. Iatrogenesis^1,3^ 4. Falling through the gaps^1,3,4^	1. My problems are my problem^3,4^ 2. The ideal patient^1,4^	*Behavioural* 1. Escaping Treatment^2^ 2. Refusal^2^ *Cognitive* 1. Compariso^2,4^ 2. Resignation^2^

Note. Findings are consistent with Research Questions as follows: ^1^ How do organizational practices, policies, and protocols complicate youth mental health care seeking? ^2^ How do youth cope with barriers if and when encountered? ^3^ In what way do youths’ conceptualization of their difficulties change in response to barriers? ^4^ How are youth understandings of mental health treatment shaped by their experiences?

### Organizational Moment: Standardization of Practices

Analysis of youth interviews revealed that standardization of practices that included strict policies and protocols dictating treatment pathways led to recurring complications among youth. The ways in which standardized care practices complicate youth mental health journeys are reviewed below, along with related behavioral and cognitive coping strategies and resulting schematic shifts in how youth come to understand their own mental illness, treatment, and the care system. Relevant research questions are indicated in headings if youth comments relate to those questions.

### Complications Experienced by Youth

#### Meeting Criteria (RQ1)

Although using standardized treatment assignment practices based on specific criteria (e.g., diagnosis) are found to be generally effective (see Cook et al., 2017), many youth expressed frustration and complications related to their treatment assignment not accounting for other key individual factors. Hayleigh voiced concerns relating to her and others’ admission to an intensive outpatient program based predominantly on her diagnosis:

They say it’s 15 and up, all ages, but they’re not. They’re centred on teenagers, and I am no longer 15 years old. (…) The [programming] is more catered towards younger people who are in high school. Also, there were two guys in when I was there. And like, they were not treated the same as the girls in the program. The support just wasn’t there for them even though they were let in. (Hayleigh, 21 years old)

Hayleigh believed that the program indicated for her, based on her diagnosis, was inappropriate given her and others’ specific circumstances (i.e., age, gender), which left her frustrated and lacking confidence in the program. Several youth shared her disillusionment with diagnosis playing such a significant role in the dictation of appropriate treatment. Trevor described his reaction when confronted with restrictive treatment options for his condition:

OCD [obsessive compulsive disorder] is a tough disorder (…) it’s very personal in terms of there’s not really anything that works the same for everyone. It’s kind of you approach it on a case-by-case basis. (Trevor)

For Hayleigh and Trevor, diagnosis or rigid criteria were perceived to be poor solitary metrics for determining treatment given limited consideration of pertinent individual factors. An additional subset of youth described limitations in being able to obtain a diagnosis to meet the threshold for admission to a program. Alise described inherent flaws in the system:

People who can make a diagnosis, it normally costs money. If people who are students or people who can’t go through their parents to do mental health things then they can’t do that (…) If you can’t get diagnosed, then you can’t necessarily solve the problem. (Alise)

Evident in her account is the impact that standardized approaches have on youth seeking mental health support when those approaches rely on restrictive criteria. A variety of obstacles to obtaining treatment were described as stemming from their marginalized position, and treatments were often ineffective or insufficient due to limited consideration of other relevant factors.

#### Partial Help (RQ1, RQ3)

When treatment assignment based on standardized metrics did result in improvement, many youth described complications stemming from only partial effectiveness of interventions (i.e., functioning improving in certain areas but not others). Treatments are often evidence-based for specific diagnoses and must be administered to fidelity, leaving little room for providers to focus on symptoms or concerns outside the treatment’s specific aims. Lindsay described her struggle with receiving treatment focused exclusively on her depression despite presenting with four diagnoses (i.e., generalized anxiety disorder, disordered eating, obsessive compulsive disorder, and major depressive disorder):

That’s probably what people have helped me with most and like focused on most [depression]. But maybe it’s just because I am depressed and always feeling trapped, but I feel like nothing’s helping (…) I mean, they could be helping, it could be beneficial. Maybe I just don’t see it or feel it. (Lindsay)

Lindsay’s account is like those of many youth who expressed difficulty with perceiving improvement in their functioning when treatment was directed only at a single set of symptoms. Treatments are occasionally designed to target symptoms associated with multiple diagnoses (e.g., depressed mood and anxiety), but such transdiagnostic interventions are not widely used and research examining their effectiveness is promising but still emerging (Bähr et al., 2025; Carlucci et al., 2021; Martin et al., 2018).

#### Iatrogenesis (RQ1, RQ3)

Decreased functioning or worsening of symptoms due to a treatment intended to lead to improvement is referred to as iatrogenesis (Mash & Wolfe, 2018). A subset of youth whose treatment was complicated by strict treatment assignment policies identified a perceived decrease in functioning due to the treatment they received. Emily described the negative impact from being involved in a group treatment for disordered eating:

I did group therapy at one point, which, I don’t know if group therapy for anorexia is a great idea because it kind of (…) it sounds like, “She ate less than me this week.” Like, you know, it becomes the whole competitive nature of the illness. (Emily)

She reported believing that the treatment format (i.e., group as opposed to individual) contributed most significantly to the iatrogenic nature of the support she received. In comparison, several youth expressed frustration with treatment modalities, rather than delivery format, worsening their condition. Madelyn described her frustration with providers cycling through multiple approaches:

I feel, like, I’m being tested all the time, like being, like, prescribed these different drugs and being prescribed meal plans and they constantly say, like, “No-one really knows anything about this disorder and it’s a huge mystery,” and I just literally feel like an animal in a cage being tested. And they’re, like, “Well try this—oh, that makes everything ten times worse? Well, let’s try this. Oh, that makes everything ten times worse too? Let’s try this”, and nothing of it really ever helps. (Madelyn)

Youth accounts exhibit ways in which narrow inclusion criteria for treatment assignment may contribute unintentionally to a reduction in functioning and wellness. Rigid approaches dictating assignment of treatment modality or format often overlook individual factors that can complicate the mental health journeys of youth.

#### Falling Through the Gaps (RQ1, RQ3, RQ4)

Many youth described the ways in which standardized care assignment approaches ultimately led to them falling in between or outside available services with no clear indication of a path to treatment. Gretchen described her time in limbo while trying to access support for her eating disorder:

They [emergency room providers] were like, “You lost too much weight. You’re not medically stable, but you’re not sick enough to be in the hospital. You’re too sick for us to offer you services, but there’s nothing else we can do. Go home, and if you get your weight up, you can come back.” (…) The first day back in [city], I went to [addictions/mental health clinic], I got an intake interview. They called me in, and they said, “You’re too sick, we don’t believe you’re medically stable, so we’re not going to offer you any service until you go to a GP [general practitioner].” (Gretchen).

Her journey involved cycling through options that were unable to provide her with treatment, with referrals and recommendations leading repeatedly to dead ends. Challenges relating to not meeting criteria for admission to programs were common among youth. According to Sonya:

I went to the interview which took three weeks for them to get back to me, first of all, to get that stupid interview, and then after the interview, they called me and were like, “Yeah, your conditions are too bad, like you” – blah, blah, blah. “I’m sorry, like, we can’t admit you.” And I was like, “What?” Like they’re supposed to help me and then they say “No” because [my condition] was like too advanced. (Sonya)

Youth often found themselves without alternative options when denied access to support due to a mismatch between symptom acuity and available supports. Although “falling through the gaps” was a common occurrence identified by youth, a subset of youth identified gaps associated with aging out of services. Brendon shared his age-related experience and concerns:

My addictions counsellor support worker – I’ll have to stop seeing her and I’ll have to stop seeing [psychiatrist], which isn’t necessarily a problem for me but once I turn 19 (…) I will never understand why. I’ll just forever be baffled by the way society looks at age. (Brendon, age 18)

Brendon highlighted the challenges inherent in an underserviced system that provides age-specific treatments. With youth facing challenges in accessing services and navigating inconsistent and variable gaps between potential supports, the “aging out” aspect of youth mental health treatment presents a significant barrier to continuity of care between services or providers. Overall, youth identified complications aligned with each step of what would be a typical treatment journey (i.e., admission, treatment, and discharge). Their accounts suggest that each step can present unique complications to mental health journeys.

### Schematic Analysis

Youth evoked various schemata during interviews, drawing on their understanding of standardized policies and practices to help make sense of the ways in which their journeys were complicated. Schemata included *my problems are my problem* and *the ideal patient*.

#### My Problems Are my Problem (RQ3. RQ4)

Youth frequently spoke about “their problems” and what they represented within the care system. It was evident in youth accounts that barriers associated with strict inclusion criteria led them to reconceptualize what their illness represented. Rather than as an indicator of treatment need, type, and intensity (as is the perspective of the Organization and the goal of standardized approaches to care assignment), youth felt that their problems were their own. Gregory spoke at length of his difficulty accessing supports and feeling disincentivized to disclose his difficulties to a system that he perceived was not able to assist him:

I don’t see what’s the help in it. I mean, yeah, I can sit there and tell them [service providers] about what happened. So what? I just told them about what happened. What are they going to do about it? (…) If I want to deal with it, I’ll deal with it. (Gregory)

The tendency to take ownership and responsibility of presenting problems and recovery was present in several youth accounts and, as with Gregory’s, typically related to a perception that the Organization could not meet their unique needs. Mia noted the same, stating, “You’re wasting your time by sitting there for an hour of your life or how many days you do it by sitting there talking to somebody when you could be out doing something else and making yourself better.”

While some youth saw little value in disclosing their difficulties to providers, particularly after experiencing prolonged challenges accessing care, others saw them as appropriate recipients of their disclosure but still not able to be relied upon for support. Interestingly, this led to youth conceptualizing their problems as “problems” from their own perspective while believing that they would simply be perceived as a “cool story” by the Organization (i.e., their issues would not be treated as a problem). Both Paul and Arabella described this phenomenon, respectively:

The way I look at it is basically my problems are my problems. I’ll tell somebody [in reference to service providers] about it, like, if it’s a cool story or something. But I’m not just going to complain to them about why my life’s shit. (Paul)

I don’t like telling people. I have a cool story, I’ll tell people. Yeah, like you do kind of feel better after you’ve told somebody about it, but, like, that’s all you get is just, like, that little bit of weight off your chest because you just talked to somebody. But that’s not solving that problem. (Arabella)

The Organization is perceived to be incapable of addressing youth difficulties due to youth conceptualization of their own problems as uniquely theirs to manage. When the Organization is viewed as an oppressive structure that rigidly dictates treatment based on certain factors (as exemplified by the above noted complications), youth turn their focus inward as it relates to improving functioning.

#### The Ideal Patient (RQ1, RQ4)

Youth discussed their symptoms and response to treatments in comparison to what they perceived the Organization expected. The “ideal patient” symbolized this comparative entity, with youth expressing frustration in being conceptualized in unrealistic terms. Brin maligned *the ideal patient*:

They just wanted to take all these perfect little pieces of what humans are supposed to be and stick them on top of my disease. And just make me into this measured, calculated idea of what a healthy person is. They say this is why you’re feeling this and when you feel this way this is what you have to do to change that and if you keep doing that then you’ll get healthy. (Brin)

Youth felt they could not possibly see success in the treatment regimen presented to them because they did not perceive themselves to align with the ideal patient meant to benefit from the Organization’s dictated approach. Cherice critically evaluated the restrictive approach to treating an “ideal patient”:

How do you take a group of people who are suffering from such an evil disorder and, like, all eating disorders not just anorexia nervosa, that have so many (…) causes and contributing factors? So many different manifestations and treat it with one program and one protocol (…) So, I don’t think there’s one standardized way of treating this disorder. (Cherice)

Youth evoked the schema of *the ideal patient* to make sense of the ways their journeys were complicated by unrealistic comparisons to ideal norms—comparisons that left them feeling powerless and unable to meet treatment expectations.

#### Coping Strategies (RQ2, RQ4)

Youth highlighted the use of two behavioral (i.e., *escaping treatment*, *refusal*) and two cognitive (i.e., *comparison*, *resignation*) coping strategies to navigate complications resulting from the Organizational Moment. Strategies were predominantly focused on avoidance of treatment options that youth felt were inappropriate or unhelpful, or acceptance of their situation as imperfect but unavoidable.

#### Behavioral Coping

##### Escaping Treatment 

Participants frequently misrepresented their situation or wellbeing to avoid or “escape” treatment. Motivations to employ this behavioral strategy were varied. Aden noted:

I remember feeling uncomfortable and I hated it. It made me feel awful being there, being around all these other people who are sick. And no, it wasn’t a good experience of mine, being there (…). I’m good at lying my way out of things, (…) so I just told them, “I’m feeling better. I want to get out of here.” And so, I was out. (Aden)

Aden became uncomfortable with the treatment process and felt it was contributing to a decline in his functioning, which motivated him to escape the situation through misrepresentation of his wellness. Aden’s experience is representative of initial contact with services, but many youth described regularly making use of escape strategies. Alyssa described her interaction with a long-time provider:

I was just kind of going, saying, “Yeah, this is good. Whatever, whatever. My meds are great.” ‘Cause you know what? I didn’t want to sit there, and I didn’t want to talk about my problems. (Alyssa)

Youth generally felt the need to behave in ways that would allow them to escape services or interactions they perceived to be unhelpful.

##### Refusal 

As a precursor to *escaping treatment*, youth made use of various refusal techniques to avoid engaging in treatment they believed would be unhelpful. In other instances, youth refused to continue engaging in treatment after determining it was ineffective. According to Joselyn:

We went to my doctor to get more medication or something, I don’t know. I didn’t take it, obviously. I was just like, “No I’m not taking that” (…). I was like, “This is not helping; it’s making me eat more.” It was making me not care, so then I would just be like, “Oh I don’t care, like, I’ll just eat anyway.” (Joselyn).

In contrast to Joselyn’s experience, some youth engaged in refusal prior to beginning a recommended treatment due to their perception that it would be ineffective. Ryana exemplified how she felt alternative methods would be more effective than those being offered:

They were, like, coming up with these pills and they’d make me not angry and everything. And I’m just like, “No; it’s just self-control. I’ve been doing it all my life.” They were just, like, straight for the pills, basically. (Ryana)

It is worth noting that Ryana showed insight into her difficulties and potential solutions, and, in addition to refusing the offered treatment, she emphasized that alternative treatments could be more beneficial. Youth who engaged in *escaping treatment* strategies tended to express discomfort with the support they were receiving and a desire to escape that discomfort. In contrast, youth who engaged in *refusal* strategies tended to express dissatisfaction with the potential effectiveness of supports and often felt other methods could be more effective.

#### Cognitive Coping

##### Comparison 

Youth frequently found solace in comparing their situation to that of others in an apparent attempt to minimize their own complications in making use of mental health supports. Lena encapsulates the use of comparison as a coping strategy:

I think everyone experiences their mental health journeys different. I know people who have suffered from eating disorders. I know people who have recovered within like three years, four years. I know people who’ve had them for like eighteen years and they’re still not recovered. I know people who are recovered but still struggle. I think for me I think I got a good one [mental health journey] (…) I’m not fully recovered, but, I’m getting there (…). Hopefully, I’ll recover at some point. (Lena)

Lena was not alone in using comparison to others to make sense of her complications. Liza described how she compared her own needs to those of others when faced with ineffectual support:

It’s not the same for everybody. I know people that need to be pushed more and so I think it’s hard to map out a great plan because I know that every single person needs different help, and they need different people helping them and they need a different plan in order to fit how they are. (Liza)

**Resignation.** Participants adopted a sense of “resignation” in response to the continued complications faced in accessing and receiving care from public supports. They appeared to arrive at the conclusion that their journeys were destined to be dissatisfying due to their conceptualization of the Organization as rigid in its approach. Felix likened the experience to other government services commonly considered to be challenging to access:

Everyone will have shitty experiences with government-run institutions (…) I think there’s just a natural kind of perspective that it’s never going to be easy. Like, the “DMV” [Department of Motor Vehicles], no one is ever going to the “DMV” just cause. It’s – it’s always going to be a pain in the ass. (Felix)

Robynne expressed a similar sense of hopelessness, resigning herself that accessing services would never be easy:

It’s so hard to get services. It’s so hard to get referred. It’s so hard to have to go into [city] every week to receive the services. It’s so hard to actually get admitted because they never admit you unless you’re about to die. (Robynne)

Her frustration with accessing services, in part due to overly rigid assessment criteria (i.e., “they never admit you unless you’re about to die”), manifested in a general cognitive assumption that the system would always present barriers to access.

## Discussion

We examined the role that restrictive standardized public health care policies and protocols play in complicating youth mental health care seeking and treatment. Youth accounts elucidated how their marginalized position in the care system affected their ability to navigate and benefit from overly restrictive approaches. A variety of negative effects on youth mental health journeys were revealed (e.g., overreliance on narrow criteria to dictate care paths, variable impact on symptom progression, abrupt disconnection from support). Opportunities for greater youth engagement and improved adherence to treatment recommendations were also revealed.

Consistent with RQ1, the marginalized position youth occupy left them at particular risk for complications related to standardized approaches to care assignment when those approaches were restrictive. Based on youth accounts, they experienced challenges with criteria or diagnosis dictating their treatment, progressed through treatment in ways that either failed to improve symptoms or worsened functioning, or were inappropriately disconnected from services before adequate stabilization. These experiences left youth feeling dissatisfied and disillusioned with the system designed to provide care. This dissatisfaction likely reduces willingness to continue seeking support (see Radez et al., 2021) and aligns with Andersen’s (1995) behavioral model of health care use. The decrease in willingness may be compounded among youth due to the unique challenges they face in identifying need, initiating contact, and accessing affordable services (Birleson & Vance, 2008; Tilleczek et al., 2017).

Despite challenges, youth employed both behavioral and cognitive coping strategies to manage these barriers (RQ2). Youth often engaged behaviorally in *escaping treatment* by minimizing or misrepresenting their distress to avoid unsatisfactory care or *refused* treatments they perceived as ineffective or misaligned with their needs. This form of expressive suppression is consistent with emotion regulation strategies observed among adolescents, particularly when social support is limited or stigma is anticipated (Larsen et al., 2012). Such suppression may be protective in the short term but is linked to long-term emotional strain and reduced openness in care relationships. Youth also used cognitive strategies, including *comparison* to others’ experiences as a way to normalize their struggles and sometimes adopted a stance of *resignation* toward the anticipated difficulties in accessing and benefiting from care. These cognitive strategies are well documented within Social Comparison Theory, which posits that individuals engage in both upward and downward comparisons to evaluate their own experiences (Festinger, 1954). While downward comparisons may momentarily alleviate distress, upward comparisons can exacerbate perceptions that their problems are inadequate to receive support and further disconnection from care systems (i.e., resignation; Buunk & Gibbons, 2007).These strategies appear broadly to reflect youth efforts to retain some control and self-protection within a system that often fails to meet their individual needs.

Related to RQ3, barriers collectively appeared to contribute to shifts in how youth conceptualized their mental health difficulties. Many internalized the belief that “my problems are my problem,” perceiving their struggles as uniquely theirs to manage without support from the care system. This may be exacerbated by internalized stigma stemming from societal beliefs that needing help is a personal weakness or failure. Longitudinal research indicates that internalized stigma predicts greater secrecy and isolation among adolescents with mental health challenges (Kaushik et al., 2016; Sivaratnam et al., 2024). Youth also negatively compared themselves to an “ideal patient”—a representation of an unrealistic “perfect” patient who fit neatly into a standardized category. This notion aligns with labeling theory, which suggests that once youth are labeled (or perceive themselves as not matching the ideal label), they may anticipate judgment or rejection, leading to avoidance or disengagement from services (Link et al., 1989; Scheff, 1974). These processes further diminish trust in the system’s ability to support recovery, reinforcing feelings of alienation.

Overall, and related to RQ4, youth understandings of mental health treatment were shaped profoundly by their lived experiences. Repeated encounters with rigid eligibility criteria, exclusion from services, and trial-based treatment modalities appeared to contribute to skepticism about the appropriateness and efficacy of available supports. Youth recounted being deemed “too sick” or “not sick enough” for care and experiencing worsening symptoms due to treatment approaches that overlooked individual complexities. Over time, these experiences contributed to perceptions of treatment as ineffective or untrustworthy, further reducing youths’ willingness to engage and increasing hopelessness. Extant literature emphasizes that stigma and rigid “sick-enough/ not-sick-enough” thresholds can create barriers that discourage continued engagement (Rose et al., 2023).

**Fig. 1:**
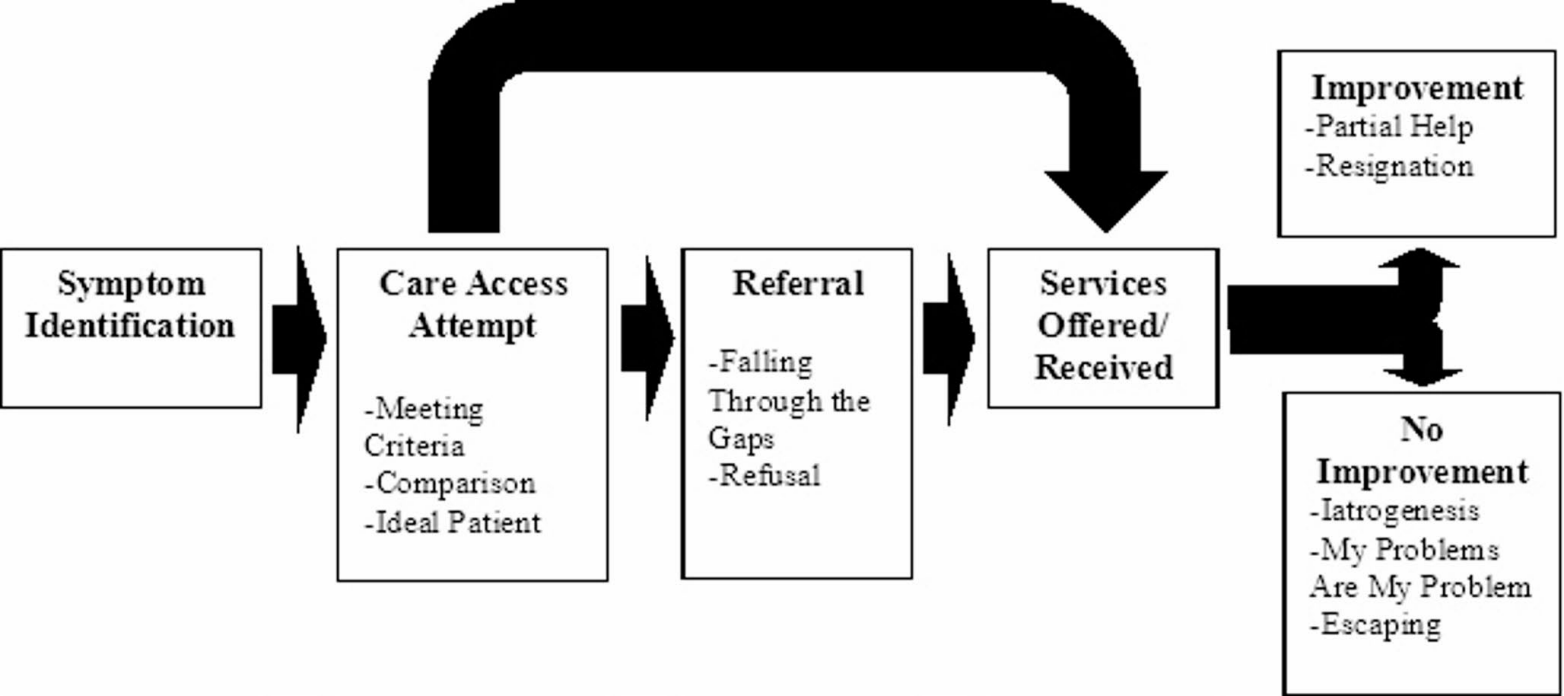
Potential impact of the Organizational Moment on youth care seeking

The ways in which the components of the OM might affect an individual are varied. However, it is likely based on youth accounts that many individuals feel the impact at multiple junctures throughout their care seeking and treatment journeys. Figure 1 presents an illustrative narrative on how an individual or individuals might be affected at various stages.

As shown, a youth might attempt to access care only to struggle with meeting the criteria for treatment acceptance. This might lead them to shift their understanding of what a mentally ill youth looks like (i.e., the ideal patient) and engage in cognitive coping by viewing their situation as better off compared to other youth. Next steps may include an offer of treatment, or referral to treatment which presents the possibility that the youth may get “lost” in the transfer or refuse inappropriate referral options. Should services be received, improvement might lead to the youth receiving only partial help for their problems and resignation that full improvement is not possible. Non-improvement might mean a worsening of symptoms due to the treatment (i.e., iatrogenesis), or an increased belief that the youth must solve their own problems because the Organization is not able due in part to dissatisfaction with the system.

Based on youth accounts, increasing satisfaction with treatment relied on improving perceptions of appropriateness and effectiveness. These perceptions are often fostered through ongoing communication and progress monitoring (Birleson & Vance, 2008; Tasca et al., 2019). Unfortunately, overly restrictive and standardized approaches frequently prioritize regulations and fidelity to protocols over individualized progress monitoring (Martin et al., 2011). Ultimately, expecting youth to self-advocate in this context to obtain this information conflicts with their developmental capacities (Birleson & Vance, 2008; Tilleczek et al., 2017), thereby maintaining their marginalization.

Extant literature and strategic directions from the Mental Health Commission of Canada (2016) emphasize the need for youth engagement and collaborative partnerships to enhance advocacy and incorporate youth perspectives (Akiva et al., 2014; Levac et al., 2019; Zeldin et al., 2013). However, youth engagement in research and service redesign is often lagging, due to institutional, ethical, and practical barriers (Health Expectations, 2022). Youth in this study described complications arising from deficits in dissemination, research, collaboration, and leadership opportunities, which were key areas identified but not yet fully realized within current systems. Addressing these gaps is essential to improve youth experiences, reduce barriers, and foster more adaptive youth-centered mental health care.

### Benefits To the Institution

Standardization of mental health treatment practices is intended to benefit both the institution and the individual. However, the extent to which practices benefit the institution is discordant with the benefit to the individual and, in some instances, further complicates youth mental health journeys.

#### Economy of Staffing

Allowing restrictive criteria (e.g., diagnosis, symptom acuity) to dictate treatment pathways offers economic efficiency by enabling front-line providers (e.g., social workers, counsellors, youth workers) to deliver standardized treatments across diagnoses, assuming clients are correctly assigned to treatment streams. This allows more highly trained and higher-paid professionals (e.g., psychiatrists, psychologists) to focus on supervision and complex cases, reducing their direct treatment roles and overall staffing costs (Martin et al., 2011). As a result, services are delivered more widely at lower expense, with fewer highly skilled positions required. Youth mental health journeys are complicated by the drive to cement this organizational structure in that they run an increased likelihood of a mismatch between their treatment needs and the competency of their providers due to restrictive classification criteria that may not appropriately capture their needs. This appeared to be the case for youth in this study, with complications stemming from two associated issues:

1. If the criteria related to demographics, diagnosis, symptom acuity, or complexity were not met, youth were not provided with alternative treatment option regardless of the fit or appropriateness of their current treatment/provider, and;
2. If deviation from a standard treatment stream or assignment was indicated, broad challenges with underservice resulted in fewer highly trained providers to deliver that care or provide necessary consultation.

#### The Empirical “Shield”

Application of restrictive and standardized approaches to mental health service delivery is often accomplished through empirically supported models for care assignment and treatment delivery. These are typically earmarked unconditionally at a certain point as effective in improving patient functioning (Drake et al., 2001; Martin et al., 2011). The empirical support for these approaches likely provides the Organization a degree of protection from criticism or critical evaluation of the practices, which can complicate mental health journeys for youth in two problematic ways:

1. Provincial mental health services may rely on the pedigree of their approaches and practices as assurance for improved patient outcomes rather than employing ongoing progress and outcome monitoring tools or monitoring additional outcome metrics, and.
2. Any public scrutiny of evidence-based practices may be defended fervently with general empirical and theoretical support.

Ultimately, this potentially increases likelihood of the Organization to conduct business as usual, without intrinsic motivation to critically evaluate their own practices on an ongoing basis. Barriers to care identified in youth accounts suggest that ongoing evaluation of standardized clinical efficacy and effectiveness is needed to promote optimal outcomes.

### Implications and Policy Recommendations

Youth described a multitude of complications in accessing and receiving care for mental health difficulties that were the result of restrictive standardized policies and practices dictating support decisions. Addressing identified complications in youth mental health journeys will be largely dependent on efforts by public mental health services to increase engagement, collaboration, and critical review of practices.

#### Recommendations

P-SEC is rooted in theory and practice that serves to examine the “relations of ruling”—policies and practices by overarching institutions that benefit the institution while disproportionately complicating the lives of marginalized individuals (Gouliquer & Poulin, 2005). By directly examining youth perspectives on restrictive and standardized approaches to care delivery and the resulting impact on their mental health journeys, the current findings are strongly positioned to influence change at a policy level. Although standardized care assignment practices stand to result in benefits for timely, appropriate, and equitable services for youth, noted complications should be considered in improving existing approaches.

##### Transdiagnostic approaches 

Mental illness diagnoses, as defined by major classification systems such as the *Diagnostic and Statistical Manual of Mental Disorders* and the *International Classification of Diseases* (American Psychiatric Association, 2022), are largely categorical. In this way, they represent an attractive (and sometimes restrictive) way for the Organization to dictate care pathways. However, psychology is shifting toward more dimensional understandings of mental illness that better reflect symptom complexity, comorbidity, and variation across the lifespan (Haltigan et al., 2020). In response, transdiagnostic treatments have emerged to target shared symptoms across disorders, such as emotional avoidance or dysregulation common in anxiety and depressive conditions (Barlow et al., 2017).

These approaches are especially beneficial for youth, who may present with overlapping or subthreshold symptoms that fall outside traditional diagnostic boundaries (Carlucci et al., 2021; Martin et al., 2018). The current study found that many youth viewed the use of diagnosis as a classifier to be a limiting factor in accessing care. A shift toward transdiagnostic models (e.g., the Unified Protocol [Barlow et al., 2017]) could support more flexible, individualized treatment while allowing the Organization to face fewer limitations related to identifying appropriate treatment streams.

##### Standardized progress and outcome monitoring 

Progress and outcome monitoring is a widely recommended component of evidence-based mental health care (American Psychological Association (APA), 2006; Tasca et al., 2019). Research shows that clinicians who regularly use monitoring tools achieve better outcomes than those who do not (APA, 2006; Jensen-Doss et al., 2018; Tasca et al., 2019). Numerous validated and accessible tools exist (e.g., Outcome Questionnaire-45; PROMIS measures) that can be implemented across treatment settings, often with minimal cost and training. These tools can be used both within treatment (e.g., as pre/post measures) and beyond symptom tracking to assess broader domains like functioning and therapeutic alliance as youth are supported in accessing treatment. Standardized monitoring also supports youth engagement. In this study, youth commonly described a lack of collaboration and limited ability to provide feedback. Research has shown that involving clients in tracking their own progress fosters greater empowerment, satisfaction, and alliance with providers (Tasca et al., 2019). Barriers to implementation, like provider knowledge gaps, concerns about misuse for performance evaluation, or skepticism about validity, can be mitigated through education and transparency to significantly improve adoption (Tasca et al., 2019).

### Limitations

Despite the study’s broad scope for a typically hard-to-reach population, several limitations must be acknowledged. First, participants’ experiences reflect policies specific to their region, potentially limiting the transferability of findings. While PEI’s public health services were the organizational focus due to similarities to other Atlantic provinces and comparable regions, geographic differences remain. For instance, as this study centered on youth in largely rural or semi-rural, underserviced areas, future research should explore barriers in urban or more densely populated settings. Second, the data reflect individual youth perspectives, offering only one view of a complex treatment dynamic. Although these perspectives are valuable due to youth’s marginalized status within systems of care, incorporating voices of parents and caregivers would enhance understanding. Prior research underscores the critical role caregivers play in facilitating access to services (Birleson & Vance, 2008; de Haan et al., 2013; Tilleczek et al., 2017), suggesting their inclusion would add important context. Third, P-SEC’s analytical framework emphasizes systemic complications in youth care, which may result in a focus on negative experiences. Future studies using different methodologies could examine benefits associated with standardization of practices. Finally, the age of the data may impact generalizability. Replication in different timeframes or contexts would help assess the stability and relevance of these findings.

#### Generalizability

The generalizability of this study’s findings is limited by the age of the data, which were collected approximately 10 years ago. Since then, mental health service delivery has changed in many ways, particularly with the rise of virtual and telehealth platforms stemming from the COVID-19 global pandemic. Stepanova and colleagues (2024) note that, although some studies report improved access and flexibility, other research suggests increased care disparities among marginalized groups, including youth. The pandemic’s impact varied by region and organizational capacity, with some areas maintaining services and others experiencing lasting declines (Campion et al., 2022). These shifts suggest that many of the barriers identified by youth in this study likely remain relevant. Marginalized populations, such as youth as conceptualized in this study, appear to have been disproportionately affected by service changes. Thus, the findings likely continue to reflect broader challenges in youth mental health care today.

## Conclusion

The benefits inherent in standardization of mental health treatment practices come at the expense of complications to youth mental health journeys with disproportionate benefits to public mental health. When facing complications, youth are forced to cope in various ways (e.g., misrepresenting symptom presentation and severity, resigning themselves to accept support they believe to be inadequate). In general, these experiences appear to leave youth feeling as though their problems cannot be addressed by the system, and in some cases, that they are left to support themselves. However, modifications and additions to existing approaches across therapeutic environments stand to greatly increase the benefit to youth making use of these services. As was exemplified in Fig. 1, opportunities likely exist at many points within youth mental health journeys to bring about change. Efforts to implement recommendations at a practice and policy level, combined with increased research related to the identified organizational factors, would lead to a cumulative improvement in youth care seeking. With an increased focus on individual factors and an intentional shift toward treatment approaches that can account for variations in symptom presentation among diagnoses, a greater impact can be made on youth struggling with mental illness.
